# Boys and Girls on the Playground: Sex Differences in Social Development Are Not Stable across Early Childhood

**DOI:** 10.1371/journal.pone.0016407

**Published:** 2011-01-28

**Authors:** Stéphanie Barbu, Guénaël Cabanes, Gaïd Le Maner-Idrissi

**Affiliations:** 1 Laboratoire EthoS - Ethologie animale et humaine, UMR 6552-CNRS, Université de Rennes 1, Rennes, France; 2 Centre de Recherche en Psychologie, Cognition et Communication, Laboratoire de Psychologie du Développement et de l'Education, Université de Rennes 2, Rennes, France; University of Liverpool, United Kingdom

## Abstract

Sex differences in human social behaviors and abilities have long been a question of public and scientific interest. Females are usually assumed to be more socially oriented and skilful than males. However, despite an extensive literature, the very existence of sex differences remains a matter of discussion while some studies found no sex differences whereas others reported differences that were either congruent or not with gender stereotypes. Moreover, the magnitude, consistency and stability across time of the differences remain an open question, especially during childhood. As play provides an excellent window into children's social development, we investigated whether and how sex differences change in social play across early childhood. Following a cross-sectional design, 164 children aged from 2 to 6 years old, divided into four age groups, were observed during outdoor free play at nursery school. We showed that sex differences are not stable over time evidencing a developmental gap between girls and boys. Social and structured forms of play emerge systematically earlier in girls than in boys leading to subsequent sex differences in favor of girls at some ages, successively in associative play at 3–4 years, cooperative play at 4–5 years, and social interactions with peers at 5–6 years. Preschool boys also display more solitary play than preschool girls, especially when young. Nevertheless, while boys catch up and girls move on towards more complex play, sex differences in social play patterns are reversed in favor of boys at the following ages, such as in associative play at 4–5 years and cooperative play at 5–6 years. This developmental perspective contributes to resolve apparent discrepancies between single-snapshot studies. A better understanding of the dynamics of sex differences in typical social development should also provide insights into atypical social developments which exhibit sex differences in prevalence, such as autism.

## Introduction

Human sex differences are a perennially hot topic that not only grips the public interest, but that has triggered a great deal of scientific focus from biological to social sciences. One of the many, and perhaps most striking, paradoxes of gender studies is that, despite decades of concerted efforts, the very existence of sex differences remains debated [1]–[3]. Discrepancies between studies undoubtedly feed the continuing debate. Some studies found no sex differences whereas others reported differences that were either congruent or not with gender stereotypes. Such discrepancies are especially marked in childhood. Here, we present evidence that sex differences are not stable over time. Between-sex differences appear during a limited window of development and even change direction with age. Our findings contribute to resolve the puzzling null or contradictory conclusions drawn from limited age-range samples or collapsed age-groups and raise important methodological issues such as the representativeness of samples in studies. Developmental studies are thus especially needed in order to go beyond the current debate.

One pervasive stereotype about sex-related differences is that girls and women are more socially oriented and skilful than boys and men [4]–[6]. There is some evidence in support of this view. From birth to the first year, infant females show stronger social orientation responses than infant males, with a stronger interest in human faces [7]–[8], a greater amount of eye contact [9]–[11], and more accurate imitative abilities [12]. Throughout childhood and adulthood, girls and women continue to be more socially expressive and responsive than age-matched males. Females display more emotional expression and are more skilled at decoding others' emotions [13], [14] and understanding others' thoughts [15]–[17]. They are also more prone to behave prosocially [18]. In childhood, these abilities are related to general social competence, especially in dealing with peers [17], [19], and to different interaction and communication styles that prefigure differences in women's and men's interpersonal goals [20], [21]. Finally, a variety of clinical conditions with marked social deficits, such as autism, occurs more often in males than in females, and has been described as an extreme manifestation of some male-typical traits, suggesting a continuum between typical and atypical social development [22].

Although the literature provides some empirical evidence, the picture is not as simple and univocal as described. Beyond a great heterogeneity in methodologies, whether studies found differences or not seems dependent on children's ages. Moreover, the differences reported are not especially large or consistent throughout childhood [6]. Yet the developmental dynamics of sex differences has been rarely investigated, with one notable exception, but that focused on within-sex variation rather than between-sex differences [23]. Thus, the magnitude, consistency and stability across time of between-sex differences remain questioned [5], [6], [18]. As play is at least to some extent a universal activity of childhood [24] and provides an excellent window into children's social development [25], [26] and psychosocial adjustment [27], we investigated sex- and age-related trends in social play development throughout early childhood.

Both the amount and the quality of children's play are associated with measures of social motivation and competence, in particular with peers [28]–[30]. It is well documented that with increasing age, children are more likely to engage in social play, proceeding from less to more mature forms of social interactions [25], [26], [29], [31]. However, there are also marked individual differences in the degree to which children are willing to participate in peer play [27]. Among available peer play scales, we adapted the seminal Parten's [32] framework which covers the social spectrum of children's participation in peer play, with non-social activities: unoccupied behavior (absence of focus or intent) and solitary play (playing alone or independently); semi-social activities: onlooker behavior (observing others' activity, but without entering into the activity) and parallel play (playing beside, but not with); and social play: associative play (playing with other children, but with no role assignment or organization of activity) and cooperative play (playing in organized and coordinated activities). To cover all children's social activities, we also recorded social interactions with peers when children are not playing, but are involved in sustained social exchanges (mostly conversations, which are more frequent in older children [26]), and social interactions with adults, as adults were present on playgrounds. We investigated whether girls show consistently more socially oriented and skilful forms of peer play and interactions than same-age boys from 2 to 6 years old, when most children begin to experience peer social interactions, or whether the sex difference changes as children grow older. To this end, children's play behavior was observed under naturalistic conditions at nursery schools during self-selected activities and spontaneous peer-groups.

## Results

### Developmental trends over the preschool years

**Figure 1:**
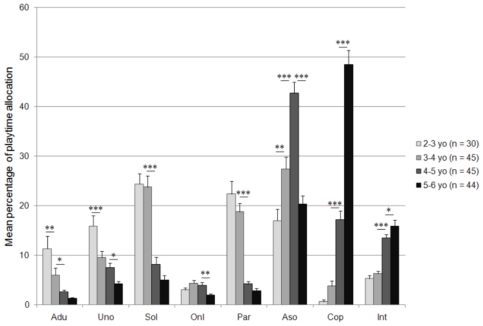
Developmental trends of children's social play from 2 to 6 years. Interactions with adults (Adu), unoccupied behavior (Uno), solitary play (Sol), onlooker behavior (Onl) and parallel play (Par) decreased significantly over the preschool years while associative play (Aso), cooperative play (Cop) and interactions with peers (Int) increased, notably with an abrupt change at 4–5 years with the predominance of associative play, and thereafter of cooperative play at 5–6 years. Bars and error bars represent mean + standard error of the percentages of children's playtime allocation within social participation categories. *P<0.05, **P<0.01, ***P<0.001 by Fisher's PLSD post hoc comparisons among age groups (see also Table S1 for complete descriptive statistics and S2 for true *P* values).

Children's social play showed important changes during the preschool period, becoming more peer-oriented and structured with age (Fig. 1; see also Table S1). We found significant effects of age for all the social categories: interactions with adults, unoccupied and onlooker behavior, solitary and parallel play decreased, while associative play, cooperative play and interactions with peers increased over the preschool years (two-way ANOVAs, all *F*_3,156_>5.2, all *P*<0.002; see Table S2). Thus, age groups were characterized by distinct social participation profiles (Fig. 1, see also Table S3). 2–3 years old children were observed more frequently playing alone or beside other peers or even unoccupied, although associative play occupied a not negligible part of their activities. They were also observed more frequently interacting with adults than older children for whom this proximity became rare. The social profile of 3–4 year olds remained quite similar to that of 2–3 year olds, except that associative play became as frequent as solitary play and more frequent than parallel play. From the age of 4–5 years, children's sociality changed abruptly, notably associative play predominated at 4–5 years and cooperative play predominated at 5–6 years.

### Sex differences over the preschool years

**Figure 2:**
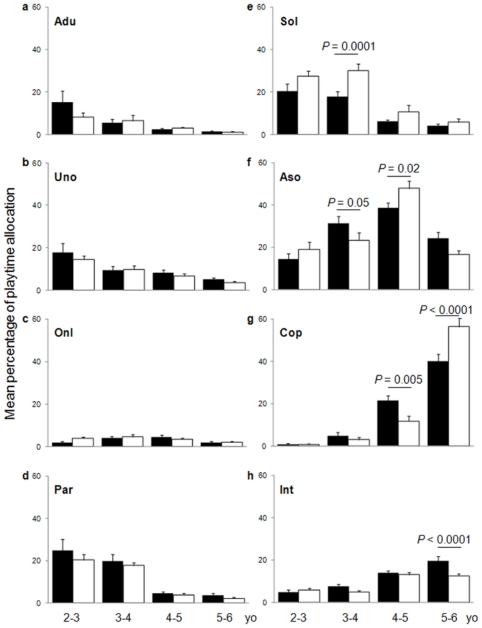
Girls develop social and complex forms of play earlier than boys, but boys catch up. Bars and error bars represent percentages (mean + s.e.m.) of children's playtime allocation within social participation categories (girls: dark bars, boys: white bars). No sex differences are found for interactions with adults (a, Adu), unoccupied behavior (b, Uno), onlooker behavior (c, Onl) or parallel play (d, Par). Sex differences appear at some ages successively in solitary play (e, Sol), associative play (f, Aso), cooperative play (g, Cop), and interactions with peers (h, Int). Significant *P* values are given for Fisher's PLSD post hoc comparisons between girls and boys within age groups. (See also Table S1 for complete descriptive statistics.)

We evidenced important sex differences in children's social play, differences that stress a developmental gap between girls and boys (Fig. 2; see also Table S1). Solitary play was influenced by sex (two-way ANOVA, sex: *F*_1,156_ = 14.30, *P* = 0.0002; age×sex: *F*_3,156_ = 2.02, *P* = 0.11): preschool boys played alone more frequently than preschool girls (Fig. 2e, top right). This difference was especially marked at 3–4 years (Fisher's PLSD, 3–4 years: *P* = 0.0001; 2–3 years: *P* = 0.08; 4–5 years: *P* = 0.15; 5–6 years: *P* = 0.59). Moreover, we found significant interactions between age and sex for associative play (age×sex: *F*_3,156_ = 4.22, *P* = 0.005; sex: *F*_1,156_ = 0.03, *P* = 0.85), cooperative play (*F*_3,156_ = 10.20, *P*<0.0001; *F*_1,156_ = 0.45, *P* = 0.50), and interactions with peers (*F*_3,156_ = 4.13, *P* = 0.008; *F*_1,156_ = 8.36, *P* = 0.004), indicating that differences between sexes changed over time. At 3–4 years, girls were involved in associative play more frequently than boys (Fig. 2f) (Fisher's PLSD, *P* = 0.05), but at 4–5 years, boys were involved in associative play more frequently than girls (*P* = 0.02). No significant differences were found in the youngest or the oldest children (2–3 years: *P* = 0.34; 5–6 years: *P* = 0.06). Sex differences in cooperative play (Fig. 2g) appeared a year later than in associative play. They appeared again first in favour of girls at 4–5 years (*P* = 0.005), but afterwards in favour of boys at 5–6 years (*P*<0.0001). No significant differences were found before these ages (2–3 years: *P* = 0.99; 3–4 years: *P* = 0.61). Thus, for both associative and cooperative play, sex differences first in favour of girls were reversed the following year. Sex differences in interactions with peers (Fig. 2h) appeared only during the final preschool year (5–6 years: *P*<0.0001; 2–3 years: *P* = 0.66; 3–4 years: *P* = 0.11; 4–5 years: *P* = 0.56), when this form of social involvement was observed gradually more frequently in girls than in boys. Finally, we evidenced neither effects of sex nor age×sex interactions for interactions with adults (*F*_1,156_ = 1.49, *P* = 0.22; *F*_3,156_ = 1.86, *P* = 0.14), unoccupied behavior (*F*_1,156_ = 1.41, *P* = 0.24; *F*_3,156_ = 0.36, *P* = 0.79), onlooker behavior (*F*_1,156_ = 0.72, *P* = 0.40; *F*_3,156_ = 1.48, *P* = 0.22), and parallel play (*F*_1,156_ = 2.42, *P* = 0.12; *F*_3,156_ = 0.27, *P* = 0.85) (Fig. 2a–d, left column).

### Girls' and boys' social profiles

To get an overall picture of sex differences, the relative frequencies of the different forms of social play at each age for both sexes must be taken into consideration (Fig. 2, see also Tables S1 and S4). At 2–3 years, the profiles of girls and boys were quite similar: children of both sexes were observed either in solitary, parallel and associative play or unoccupied in significantly similar proportions (pairewise t-tests, all *P*>0.08; except solitary vs. unoccupied for boys: *P* = 0.0003). Interactions with adults by boys were less frequent than the above activities (all *P*<0.04), but this was not so for girls (all *P*>0.20). At 3–4 years, associative play, which was more frequent in girls than in boys, was also the main form of girls' social activity (all *P*<0.04), whereas associative play was still as frequent as solitary play (*P* = 0.27) and parallel play (*P* = 0.17) for boys. At 4–5 years, although cooperative play was more frequent in girls than in boys and associative play more frequent in boys than in girls, associative play was however the main form of social activity for both sexes, ahead of the other activities (all *P*<0.001). Similarly, at 5–6 years, although girls interacted with peers more frequently than boys did, whereas cooperative play was more frequent in boys, cooperative play became the main form of play for both sexes (all *P*<0.01). Thus from 3–4 years old, girls were actually more associative than same-age boys, but in the later stages, both girls' and boys' play was mostly associative at 4–5 years and mostly cooperative at 5–6 years.

## Discussion

Our study highlights that although all children progress towards more socially oriented and skilful forms of play during early childhood, girls develop social and structured forms of play at younger ages than boys. Preschool boys also display more solitary play than preschool girls. However, boys catch up at the following developmental stages. Sex differences are not stable throughout social development, but they rather reflect a developmental gap between girls and boys. While boys catch up and same-age girls move on towards more complex social play and interactions, a sex difference recorded in favour of girls in a particular social play pattern at a given age can be reversed the following year, as we evidenced for associative and cooperative play. Therefore, it is not surprising that some studies based upon limited age-range samples or collapsed age-groups failed to find significant results or found results that were not congruent with gender stereotypes [6], making the case for more developmental studies to capture the dynamics of sex differences.

Moreover, discrepancies between studies can also be related to differences in the operationalization of sex differences and comparisons [5]. There are two ways to measure sex differences, which can provide quite different pictures of sex differences and conclusions: asking whether the behavior is more frequent in one sex than in the other or asking whether the behavior is the main form expressed by one sex compared to the other. Here, we show that, despite the advance of girls, both girls' and boys' play is associative at 4–5 years and cooperative at 5–6 years. Therefore, girls' advantage appears systematically the year before that the play activity becomes the predominant one for both sexes.

As play involves communication, role taking and cooperation, sex differences in social play may be a by-product of sex differences in socio-cognitive skills, as girls develop language [6], [33] and theory-of-mind [15]–[17] skills earlier than boys do. These sex differences may also appear during a limited window of development (during the preschool years in particular) and disappear in later ages. It is clear that there is a linkage between children's socio-cognitive skills and some aspects of social play [34], [35]. However, the relation between social play, skills and cognition must be further explored as more mature forms of play may also promote children's social and socio-cognitive skills. Play and associated interactions with peers is considered to both reflect children's social competence and to provide children with a unique environment where they can acquire important social and socio-cognitive skills [27], [28], [36]. Although there are a number of correlational studies, there is very little relevant experimental evidence, remaining open the question of cause-and-effect between play and children's skills.

Sex differences in social play patterns may also result in children's sex-typed toys and activities. Sex differences in toys and activities represent one of the largest non-reproductive physical or psychological sex differences that have been widely observed across cultures and taxa [37], [38]. Children's preferences for sex-typed toys are apparent as early as infancy [39] and increase over the preschool years [5], [6]. The context of play (e.g., play areas and materials) has significant effects on the quantity and quality of play and attendant social interactions [40]. Both girls and boys show the greatest play complexity when playing with female stereotyped toys than with neutral or male stereotyped toys [41]. Therefore, early sex differences in interests may impact upon the evaluation of children's play quality and related social and socio-cognitive skills.

The contribution of the socio-cultural and biological factors in human sex social differences is not yet known given their complex interplay [3], [38]. Many of these differences may to some extent be the result of socialization. Differences in styles of parenting towards the sexes [6] and in peer cultures within sex-segregated peer groups [42] may enhance the development of different interests and skills in boys and girls. Nevertheless, sex differences were also reported despite seemingly similar social environment and experiences suggesting a differential effect of the early environment. In particular, boys are more vulnerable to disruptive events and adverse home environments than girls [43], [44]. Sex differences at birth [7], [12] and correlations with prenatal testosterone in normally developing children (such as in eye contact [11], vocabulary size [45], and sex-typed play [46]) strongly suggest that biological factors play a role as well, at least in early sex differences. During atypical social development, foetal testosterone is also associated with the severity of autistic traits [47]. Prenatal hormonal exposure may shape the neural mechanisms underlying early social development during both typical and atypical development [22].

The questions why girls are more socially precocious than boys, and how boys eventually catch up in normally developing children, but not in children with some social developmental deficits must be studied in much depth. Understanding the developmental dynamics of relationships between social competence, social cognition and sex should provide new insights on how the nature and the weight of underlying biological and social processes change over time [48] and even between sexes [49], [50] during both typical and atypical development [22].

## Materials and Methods

### Ethics Statement

The study consisted in non-invasive and unconstrained behavioral observations of children at nursery schools during daily activities. According to the current French laws on the protection of persons in biomedical research (law No 88-1138, so-called Huriet-Sérusclat law of the 20th December 1988, amended in 2004 - law of the 9th August 2004), such protocol does not require the approval of an ethics committee. The study complies with the ethics guidelines given by the National Consultative Ethics Committee of the French Centre National de la Recherche Scientifique (COMETS). Only children, for whom parental written consent was obtained, participated in the study. The observations started after receiving written consent from the local Inspection of French National Education and permission from the schools. The data were analyzed anonymously.

### Subjects and setting

Children were selected from 16 classes in two nursery schools from urban surrounding (Rennes, France). The selection criteria were (1) that the parents provided a written consent, (2) that the child attended school fulltime, and (3) that the child age pertained to the second half of the year in order to reduce age range within age-groups and to avoid overlap between age-groups. Following a cross-sectional design, the children (n = 164: 82 boys), ranging in age from 29 to 74 months, were divided into four age groups corresponding to the four French school grades: 2–3 year olds, 3–4 year olds, 4–5 year olds, and 5–6 year olds (see Table 1 for age and sex composition of the sample). Age groups differed significantly in age (two-way ANOVA, *F_3_*_,*156*_ = 1080.93, *P*<0.0001) and contained equal numbers of children, except the youngest group as only 20% of the 2-year-old children attend school in France whereas near all children do while they are 3 years old. In each group, girls and boys (in roughly equal numbers) did not differ in age (sex: *F_1_*_,*156*_ = 0.64, *P* = 0.42; age×sex: *F_3_*_,*156*_ = 0.99, *P* = 0.40), nor they did in family backgrounds. The children were from diverse socioeconomic backgrounds (20.1% upper-class, 37.8% middle-class, 25.6% lower-class, 7.3% unemployed and 9.2% no reply).

**Table 1:** Age and sex composition of the sample.

	2–3 years old			3–4 years old			4–5 years old			5–6 years old		
	M	s.d.	n	M	s.d.	n	M	s.d.	n	M	s.d.	n
Boys	35.6	2.8	17	44.9	3.0	22	55.6	2.0	20	69.8	3.1	23
Girls	34.1	2.3	13	44.9	2.8	23	56.2	2.4	25	69.2	3.4	21
Overall	34.9	2.7	30	44.9	2.9	45	56.0	2.3	45	69.5	3.2	44

(M: Mean age in months, s.d.: standard deviation, n: number of children).

Children were observed during outdoor playtimes that occurred twice a day (morning and afternoon). Playgrounds were large outdoor areas fully equipped for children (e.g., slides, sandbox, tricycles, balls). Numbers of children in the playground varied with the size of the school (2 to 3 classes in one school and 5 to 6 classes in the other). Peer groups were mixed-aged, generally including classes from two successive grades. The adult-children ratio was approximately the same in all playgrounds and schools as teachers accompanied their classes. The teachers were in sight of the children in order to help settle any problems that might arise, but they never directed the children's activities.

### Observational procedure

The observations were made from March to May 2005 and 2006. We used scan sampling for data collection [51]. The children's activities were recorded every 2 minutes during playtime that lasted on average 30 minutes. As it was not possible to observe all the children who were present on the playground at the same time, the observer followed a same-age group of fifteen children during a session. The same number of observations was conducted for each child (i.e. 120 scans that is 4 hours of observation per child). On average, 10 free-play sessions over two weeks were needed to collect data for a group. Observation sessions were counterbalanced daily (morning and afternoon) and for a school term (beginning and end) among age groups. The daily observation order of the children was also randomized within a group. Two trained observers (both male), one in each school, collected data. They were unaware of the purpose of the study (i.e. investigation of sex differences). The observer remained visible to the children during observation sessions and adopted an integrative non-participant attitude. After a preliminary habituation period of two weeks, the observer recorded children's activities on a check sheet, using a stopwatch.

### Coding and reliability

Coding was derived from Parten's [32] peer play categories: (1) unoccupied behavior (wandering around aimlessly, watching anything of passing interest or staring off into space) (k = 0.67); (2) solitary play (playing apart from other children or playing independently without acknowledging peers playing in close proximity) (k = 0.71); (3) onlooker behavior (observing the activity of other children, within speaking distance, making eventually some comments on the activity, but with no entry into the activity) (k = 0.72); (4) parallel play (playing beside – within 3 feet, with materials that are similar to those being used by others in close proximity, but independently without substantial interaction) – in order to introduce a more clear-cut distinction between parallel and solitary play, we relied on parallel aware play [29] that is accompanied with eye-contacts and/or a few brief social exchanges (e.g., vocalization, smile) (k = 0.93); (5) associative play (being involved in similar playful activities accompanied with sustained social exchanges and following a common plan, but with a mild control of group membership and no role assignment or organization of activity) (k = 0.90); (6) cooperative play (playing in organized and coordinated activities, that is showing group membership control, division of labour and differentiation of roles, mostly enacting complementary roles within social pretend play or games with rules) (k = 0.99). We added two categories: (7) social interactions with peers when children are not playing, but are involved in sustained social exchanges (e.g., mostly conversations) (k = 0.75); (8) social interactions with adults as teachers were present on playgrounds (k = 0.95). Finally, when the target child was engaged in an activity that did not fall into the categories, mostly when he/she performed maintenance behaviors (e.g., eating a snack, going to restroom…), these scans were discarded and replaced by supplementary scans so as to have the same number of observations for each child. Before observations and coding, the two observers were previously trained on videotapes of children's outdoor free-play until they reached satisfactory inter-coder reliability. Inter-coder reliability was then established on 12 videotapes selected randomly. Cohen's kappa statistics for each social category ranged from 0.67 to 0.99 (global kappa  = 0.84).

### Statistical analyses

A proportion score was calculated for each child for each of the eight social categories based on the proportion of time intervals spent in each category (relative to total number of time intervals). Two-way ANOVAs were carried out on proportion scores to test the effects of age, sex and their interaction. When an effect was significant, Fisher's PLSD post hoc tests compared age groups or boys and girls within age groups. To assess children's social participation profiles, pairwise t-tests were used to compare the proportions of social categories. All tests were two-tailed and α = 0.05.

## Supporting Information

Table S1 **Descriptive statistics of children's playtime allocation among social participation categories within age and sex groups.** (M: Mean percentage, s.e.: standard error; Adu: interactions with adults, Uno: unoccupied behaviour, Sol: solitary play, Onl: onlooker behaviour, Par: parallel play, Aso: associative play, Cop: cooperative play, Int: interactions with peers).
(DOC)

Table S2 **Developmental trends in social participation over the preschool period.** Age effect on the percentages of children's playtime allocation among social play categories (*F* and *P*- values for variances analyses and *P*-values for Fisher's PLSD post-hoc comparisons among age groups). A main age effect was found for all the categories. More precisely, interactions with adults (Adu) showed a significant decrease from 2–3 to 4–5 years, becoming rare in the two oldest age groups. Children spent also less and less time unoccupied (Uno) with a significant decrease at the beginning and the end of the preschool period. Onlooker behaviour (Onl) which was not frequent whatever age group decreased significantly at the end of the preschool years. Solitary (Sol) and parallel play (Par) showed a similar developmental course with an abrupt decrease between 3–4 and 4–5 years. On the other hand, associative play (Aso) increased significantly between 2–3 and 4–5 years becoming twice as much frequent in 4–5 year-olds than in 2–3 year-olds, but it decreased significantly thereafter. Cooperative play (Cop) significantly increased from 4–5 years to 5–6 years, representing almost half of the children's activities at the end of the preschool period. Finally, interactions with peers (Int) significantly increased between 3–4 and 5–6 years.
(DOC)

Table S3 **Children**'**s social participation profiles over the preschool period.** Comparisons of the percentages of social play categories within age groups (pairewise t-tests: *t*- and *P*-values, *df*, and sample sizes).
(DOC)

Table S4 **Girls' and boys' social participation profiles over the preschool period.** Comparisons of the percentages of social play categories within age and sex groups (pairewise t-tests: *t*- and *P*-values, *df*, and sample sizes).
(DOC)
