# Supplementary material for: Boys and Girls on the Playground: Sex Differences in Social Development Are Not Stable across Early Childhood
Source: PLoS One. 2011 Jan 28;6(1):e16407. doi: 10.1371/journal.pone.0016407 (PMC3030576; doi:10.1371/journal.pone.0016407)
Supplement: Table S1 — Descriptive statistics of children's playtime allocation among social participation categories within age and sex groups. (M: Mean percentage, s.e.: standard error; Adu: interactions with adults, Uno: unoccupied behaviour, Sol: solitary play, Onl: onlooker behaviour, Par: parallel play, Aso: associative play, Cop: cooperative play, Int: interactions with peers). (DOC) [file pone.0016407.s001.doc]

Supplementary Table 1. Descriptive statistics of children’s playtime allocation among social participation categories within age and sex groups. (M: Mean percentage, s.e.: standard error; Adu: interactions with adults, Uno: unoccupied behaviour, Sol: solitary play, Onl: onlooker behaviour, Par: parallel play, Aso: associative play, Cop: cooperative play, Int: interactions with peers).
