# Supplementary material for: Boys and Girls on the Playground: Sex Differences in Social Development Are Not Stable across Early Childhood
Source: PLoS One. 2011 Jan 28;6(1):e16407. doi: 10.1371/journal.pone.0016407 (PMC3030576; doi:10.1371/journal.pone.0016407)
Supplement: Table S2 — Developmental trends in social participation over the preschool period. Age effect on the percentages of children's playtime allocation among social play categories (F and P- values for variances analyses and P-values for Fisher's PLSD post-hoc comparisons among age groups). A main age effect was found for all the categories. More precisely, interactions with adults (Adu) showed a significant decrease from 2–3 to 4–5 years, becoming rare in the two oldest age groups. Children spent also less and less time unoccupied (Uno) with a significant decrease at the beginning and the end of the preschool period. Onlooker behaviour (Onl) which was not frequent whatever age group decreased significantly at the end of the preschool years. Solitary (Sol) and parallel play (Par) showed a similar developmental course with an abrupt decrease between 3–4 and 4–5 years. On the other hand, associative play (Aso) increased significantly between 2–3 and 4–5 years becoming twice as much frequent in 4–5 year-olds than in 2–3 year-olds, but it decreased significantly thereafter. Cooperative play (Cop) significantly increased from 4–5 years to 5–6 years, representing almost half of the children's activities at the end of the preschool period. Finally, interactions with peers (Int) significantly increased between 3–4 and 5–6 years. (DOC) [file pone.0016407.s002.doc]

Supplementary Table 2. Developmental trends in social participation over the preschool period. Age effect on the percentages of children’s playtime allocation among social play categories (*F* and *P*- values for variances analyses and *P*-values for Fisher’s PLSD post-hoc comparisons among age groups). A main age effect was found for all the categories. More precisely, interactions with adults (Adu) showed a significant decrease from 2-3 to 4-5 years, becoming rare in the two oldest age groups. Children spent also less and less time unoccupied (Uno) with a significant decrease at the beginning and the end of the preschool period. Onlooker behaviour (Onl) which was not frequent whatever age group decreased significantly at the end of the preschool years. Solitary (Sol) and parallel play (Par) showed a similar developmental course with an abrupt decrease between 3-4 and 4-5 years. On the other hand, associative play (Aso) increased significantly between 2-3 and 4-5 years becoming twice as much frequent in 4-5 year-olds than in 2-3 year-olds, but it decreased significantly thereafter. Cooperative play (Cop) significantly increased from 4-5 years to 5-6 years, representing almost half of the children’s activities at the end of the preschool period. Finally, interactions with peers (Int) significantly increased between 3-4 and 5-6 years.

|  |  | 2-way ANOVAs | |  | Fisher’s PLSD post-hoc comparisons: *P*-values | | |
| --- | --- | --- | --- | --- | --- | --- | --- |
|  |  | *F*_3, 156_ | *P* |  | 2-3 years  vs. 3-4 years | 3-4 years  vs. 4-5 years | 4-5 years  vs. 5-6 years |
| Adu |  | 12.58 | <0.001 |  | 0.004 | 0.04 | 0.40 |
| Uno |  | 15.09 | <0.001 |  | <0.001 | 0.20 | 0.04 |
| Sol |  | 36.15 | <0.001 |  | 0.81 | <0.001 | 0.16 |
| Onl |  | 5.27 | 0.002 |  | 0.07 | 0.54 | 0.003 |
| Par |  | 48.35 | <0.001 |  | 0.09 | <0.001 | 0.47 |
| Aso |  | 29.95 | <0.001 |  | 0.001 | <0.001 | <0.001 |
| Cop |  | 148.36 | <0.001 |  | 0.24 | <0.001 | <0.001 |
| Int |  | 38.98 | <0.001 |  | 0.44 | <0.001 | 0.04 |
