# Supplementary material for: Boys and Girls on the Playground: Sex Differences in Social Development Are Not Stable across Early Childhood
Source: PLoS One. 2011 Jan 28;6(1):e16407. doi: 10.1371/journal.pone.0016407 (PMC3030576; doi:10.1371/journal.pone.0016407)
Supplement: Table S3 — Children's social participation profiles over the preschool period. Comparisons of the percentages of social play categories within age groups (pairewise t-tests: t- and P-values, df, and sample sizes). (DOC) [file pone.0016407.s003.doc]

Supplementary Table 3. Children’s social participation profiles over the preschool period. Comparisons of the percentages of social play categories within age groups (pairewise t-tests: *t*- and *P*-values, *df*, and sample sizes).

| *t*  *P* |  | Adu | Uno | Sol | Onl | Par | Aso | Cop | Int |
| --- | --- | --- | --- | --- | --- | --- | --- | --- | --- |
|  |  |  |  |  |  |  |  |  |  |
| 2-3 years old (*n* = 30, *df* = 29) | | | |  |  |  |  |  |  |
| Adu |  | - | -1.40 | -3.35 | 3.16 | -2.86 | -1.42 | 4.13 | 2.15 |
| Uno |  | 0.17 | - | -2.92 | 6.13 | -1.57 | -0.31 | 7.47 | 4.78 |
| Sol |  | 0.002 | 0.007 | - | 10.13 | 0.57 | 2.04 | 10.50 | 7.71 |
| Onl |  | 0.004 | <0.001 | <0.001 | - | -7.17 | -6.18 | 5.39 | -3.26 |
| Par |  | 0.008 | 0.13 | 0.58 | <0.001 | - | 1.40 | 7.85 | 6.14 |
| Aso |  | 0.17 | 0.76 | 0.05 | <0.001 | 0.17 | - | 7.84 | 5.99 |
| Cop |  | <0.001 | <0.001 | <0.001 | <0.001 | <0.001 | <0.001 | - | -7.63 |
| Int |  | 0.04 | <0.001 | <0.001 | 0.003 | <0.001 | <0.001 | <0.001 | - |
| 3-4 years old (*n* = 45, *df* = 44) | | | |  |  |  |  |  |  |
| Adu |  | - | -1.80 | -7.00 | 1.01 | -5.11 | -6.74 | 1.19 | -0.14 |
| Uno |  | 0.08 | - | -5.23 | 4.71 | -3.78 | -5.46 | 4.06 | 2.45 |
| Sol |  | <0.001 | <0.001 | - | 8.41 | 1.82 | -0.87 | 6.95 | 6.70 |
| Onl |  | 0.32 | <0.001 | <0.001 | - | -7.86 | -8.46 | 0.49 | -2.37 |
| Par |  | <0.001 | <0.001 | 0.08 | <0.001 | - | -2.61 | 6.51 | 6.03 |
| Aso |  | <0.001 | <0.001 | 0.39 | <0.001 | 0.01 | - | 9.08 | 9.24 |
| Cop |  | 0.24 | <0.001 | <0.001 | 0.63 | <0.001 | <0.001 | - | -3.04 |
| Int |  | 0.89 | 0.02 | <0.001 | 0.02 | <0.001 | <0.001 | 0.004 | - |

| *t*  *P* |  | Adu | Uno | Sol | Onl | Par | Aso | Cop | Int |
| --- | --- | --- | --- | --- | --- | --- | --- | --- | --- |
|  |  |  |  |  |  |  |  |  |  |
| 4-5 years old (*n* = 45, *df* = 44) | | | |  |  |  |  |  |  |
| Adu |  | - | -4.75 | -3.75 | -1.93 | -2.71 | -18.66 | -7.30 | -13.59 |
| Uno |  | <0.001 | - | -0.37 | 3.37 | 3.02 | -12.93 | -4.85 | -5.40 |
| Sol |  | <0.001 | 0.71 | - | 2.56 | 2.41 | -11.14 | -3.47 | -2.93 |
| Onl |  | 0.06 | 0.002 | 0.01 | - | -0.31 | -18.12 | -6.41 | -9.36 |
| Par |  | 0.01 | 0.004 | 0.02 | 0.76 | - | -17.64 | -6.64 | -9.76 |
| Aso |  | <0.001 | <0.001 | <0.001 | <0.001 | <0.001 | - | 7.29 | 12.46 |
| Cop |  | <0.001 | <0.001 | 0.001 | <0.001 | <0.001 | <0.001 | - | 1.89 |
| Int |  | <0.001 | <0.001 | 0.005 | <0.001 | <0.001 | <0.001 | 0.06 | - |
| 5-6 years old (*n* = 44, *df* = 43) | | | |  |  |  |  |  |  |
| Adu |  | - | -5.86 | -4.48 | -1.59 | -3.25 | -10.87 | -15.99 | -11.85 |
| Uno |  | <0.001 | - | -0.82 | 3.84 | 2.25 | -9.62 | -13.92 | -9.58 |
| Sol |  | <0.001 | 0.42 | - | 3.21 | 2.83 | -8.77 | -12.56 | -6.68 |
| Onl |  | 0.12 | <0.001 | 0.002 | - | -1.50 | -10.34 | -15.93 | -10.74 |
| Par |  | 0.002 | 0.03 | 0.007 | 0.14 | - | -10.14 | -14.39 | -9.80 |
| Aso |  | <0.001 | <0.001 | <0.001 | <0.001 | <0.001 | - | -6.65 | 1.93 |
| Cop |  | <0.001 | <0.001 | <0.001 | <0.001 | <0.001 | <0.001 | - | 9.11 |
| Int |  | <0.001 | <0.001 | <0.001 | <0.001 | <0.001 | 0.06 | <0.001 | - |
