# Supplementary material for: Boys and Girls on the Playground: Sex Differences in Social Development Are Not Stable across Early Childhood
Source: PLoS One. 2011 Jan 28;6(1):e16407. doi: 10.1371/journal.pone.0016407 (PMC3030576; doi:10.1371/journal.pone.0016407)
Supplement: Table S4 — Girls' and boys' social participation profiles over the preschool period. Comparisons of the percentages of social play categories within age and sex groups (pairewise t-tests: t- and P-values, df, and sample sizes). (DOC) [file pone.0016407.s004.doc]

Supplementary Table 4. Girls’ and boys’ social participation profiles over the preschool period. Comparisons of the percentages of social play categories within age and sex groups (pairewise t-tests: *t*- and *P*-values, *df*, and sample sizes).

| *t*  *P* |  | Adu | Uno | Sol | Onl | Par | Aso | Cop | Int |
| --- | --- | --- | --- | --- | --- | --- | --- | --- | --- |
|  |  |  |  |  |  |  |  |  |  |
| 2-3 years old: Girls (*n* = 13, *df* = 12) | | | | |  |  |  |  |  |
| Adu |  | - | -0.34 | -0.69 | 2.59 | -1.17 | 0.16 | 2.82 | 1.92 |
| Uno |  | 0.74 | - | -0.52 | 3.56 | -0.85 | 0.56 | 3.86 | 2.85 |
| Sol |  | 0.51 | 0.61 | - | 5.64 | -0.72 | 1.36 | 5.60 | 4.42 |
| Onl |  | 0.02 | 0.004 | <0.001 | - | -4.49 | -4.73 | 2.19 | -3.07 |
| Par |  | 0.26 | 0.41 | 0.48 | <0.001 | - | 1.91 | 4.60 | 3.73 |
| Aso |  | 0.88 | 0.58 | 0.20 | <0.001 | 0.08 | - | 5.84 | 4.46 |
| Cop |  | 0.02 | 0.002 | <0.001 | 0.05 | <0.001 | <0.001 | - | -5.10 |
| Int |  | 0.08 | 0.01 | <0.001 | 0.01 | 0.003 | <0.001 | <0.001 | - |
| 2-3 years old: Boys (*n* = 17, *df* = 16) | | | | |  |  |  |  |  |
| Adu |  | - | -2.80 | -5.77 | 2.07 | -4.16 | -2.22 | 3.60 | 1.04 |
| Uno |  | 0.01 | - | -4.56 | 7.33 | -1.68 | -1.14 | 10.19 | 4.69 |
| Sol |  | <0.001 | <0.001 | - | 8.66 | 1.84 | 1.52 | 9.54 | 6.42 |
| Onl |  | 0.05 | <0.001 | <0.001 | - | -6.25 | -4.33 | 5.66 | -1.77 |
| Par |  | <0.001 | 0.11 | 0.08 | <0.001 | - | 0.26 | 7.05 | 5.47 |
| Aso |  | 0.04 | 0.27 | 0.15 | <0.001 | 0.80 | - | 5.77 | 4.42 |
| Cop |  | 0.002 | <0.001 | <0.001 | <0.001 | <0.001 | <0.001 | - | -5.66 |
| Int |  | 0.31 | <0.001 | <0.001 | 0.10 | <0.001 | <0.001 | <0.001 | - |

| *t*  *P* |  | Adu | Uno | Sol | Onl | Par | Aso | Cop | Int |
| --- | --- | --- | --- | --- | --- | --- | --- | --- | --- |
|  |  |  |  |  |  |  |  |  |  |
| 3-4 years old: Girls (*n* = 23, *df* = 22) | | | | |  |  |  |  |  |
| Adu |  | - | -1.44 | -4.03 | 0.76 | -3.70 | -7.12 | 0.34 | -1.05 |
| Uno |  | 0.16 | - | -2.79 | 3.42 | -2.54 | -4.88 | 1.99 | 0.91 |
| Sol |  | <0.001 | 0.01 | - | 5.60 | -0.56 | -2.59 | 3.53 | 3.24 |
| Onl |  | 0.45 | 0.002 | <0.001 | - | -4.86 | -7.50 | -0.30 | -2.93 |
| Par |  | 0.001 | 0.02 | 0.58 | <0.001 | - | -2.20 | 3.63 | 3.21 |
| Aso |  | <0.001 | <0.001 | 0.02 | <0.001 | 0.04 | - | 7.95 | 8.21 |
| Cop |  | 0.73 | 0.06 | 0.002 | 0.77 | 0.001 | <0.001 | - | -2.03 |
| Int |  | 0.31 | 0.37 | 0.004 | 0.008 | 0.004 | <0.001 | 0.06 | - |
| 3-4 years old: Boys (*n* = 22, *df* = 21) | | | | |  |  |  |  |  |
| Adu |  | - | -1.09 | -6.21 | 0.69 | -3.50 | -3.25 | 1.31 | 0.58 |
| Uno |  | 0.29 | - | -4.74 | 3.17 | -3.05 | -2.90 | 4.40 | 2.70 |
| Sol |  | <0.001 | <0.001 | - | 7.08 | 3.41 | 1.13 | 6.91 | 6.97 |
| Onl |  | 0.50 | 0.005 | <0.001 | - | -7.74 | -4.73 | 1.29 | -0.33 |
| Par |  | 0.002 | 0.006 | 0.003 | <0.001 | - | -1.41 | 7.65 | 8.11 |
| Aso |  | 0.004 | 0.009 | 0.27 | <0.001 | 0.17 | - | 5.15 | 5.19 |
| Cop |  | 0.20 | <0.001 | <0.001 | 0.21 | <0.001 | <0.001 | - | -2.63 |
| Int |  | 0.57 | 0.01 | <0.001 | 0.75 | <0.001 | <0.001 | 0.02 | - |

| *t*  *P* |  | Adu | Uno | Sol | Onl | Par | Aso | Cop | Int |
| --- | --- | --- | --- | --- | --- | --- | --- | --- | --- |
|  |  |  |  |  |  |  |  |  |  |
| 4-5 years old: Girls (*n* = 25, *df* = 24) | | | | |  |  |  |  |  |
| Adu |  | - | -3.57 | -3.57 | -1.89 | -2.51 | -15.79 | -7.31 | -11.83 |
| Uno |  | 0.002 | - | 1.62 | 2.38 | 2.19 | -8.79 | -4.70 | -3.49 |
| Sol |  | 0.002 | 0.12 | - | 1.33 | 1.47 | -11.53 | -5.75 | -5.61 |
| Onl |  | 0.07 | 0.03 | 0.20 | - | -0.11 | -13.64 | -6.02 | -6.66 |
| Par |  | 0.02 | 0.04 | 0.15 | 0.91 | - | -13.76 | -6.52 | -7.00 |
| Aso |  | <0.001 | <0.001 | <0.001 | <0.001 | <0.001 | - | 4.09 | 9.02 |
| Cop |  | <0.001 | <0.001 | <0.001 | <0.001 | <0.001 | <0.001 | - | 3.07 |
| Int |  | <0.001 | 0.002 | <0.001 | <0.001 | <0.001 | <0.001 | 0.005 | - |
| 4-5 years old: Boys (*n* = 20, *df* = 19) | | | | |  |  |  |  |  |
| Adu |  | - | -3.38 | -2.60 | -0.62 | -1.16 | -12.27 | -3.38 | -7.56 |
| Uno |  | 0.003 | - | -1.21 | 2.46 | 2.14 | -10.22 | -2.08 | -4.27 |
| Sol |  | 0.02 | 0.24 | - | 2.26 | 2.06 | -6.12 | -0.25 | -0.62 |
| Onl |  | 0.54 | 0.02 | 0.04 | - | -0.39 | -13.52 | -3.11 | -6.46 |
| Par |  | 0.26 | 0.05 | 0.05 | 0.70 | - | -12.63 | -3.05 | -6.68 |
| Aso |  | <0.001 | <0.001 | <0.001 | <0.001 | <0.001 | - | -7.12 | 9.40 |
| Cop |  | 0.003 | 0.05 | 0.81 | 0.006 | 0.007 | <0.001 | - | -0.45 |
| Int |  | <0.001 | <0.001 | 0.54 | <0.001 | <0.001 | <0.001 | 0.66 | - |

| *t*  *P* |  | Adu | Uno | Sol | Onl | Par | Aso | Cop | Int |
| --- | --- | --- | --- | --- | --- | --- | --- | --- | --- |
|  |  |  |  |  |  |  |  |  |  |
| 5-6 years old: Girls (*n* = 21, *df* = 20) | | | | |  |  |  |  |  |
| Adu |  | - | -4.91 | -3.30 | -0.49 | -2.58 | -7.68 | -10.85 | -9.04 |
| Uno |  | <0.001 | - | 1.06 | 3.56 | 1.38 | -6.73 | -8.74 | -6.98 |
| Sol |  | 0.004 | 0.30 | - | 2.18 | 0.71 | -6.86 | -8.90 | -6.87 |
| Onl |  | 0.63 | 0.002 | 0.04 | - | -1.72 | -7.59 | -10.60 | -8.73 |
| Par |  | 0.02 | 0.18 | 0.49 | 0.10 | - | -6.80 | -9.24 | -7.07 |
| Aso |  | <0.001 | <0.001 | <0.001 | <0.001 | <0.001 | - | -2.89 | 1.06 |
| Cop |  | <0.001 | <0.001 | <0.001 | <0.001 | <0.001 | 0.009 | - | 4.55 |
| Int |  | <0.001 | <0.001 | <0.001 | <0.001 | <0.001 | 0.30 | <0.001 | - |
| 5-6 years old: Boys (*n* = 23, *df* = 22) | | | | |  |  |  |  |  |
| Adu |  | - | -3.46 | -3.38 | -1.86 | -2.00 | -9.03 | -13.78 | -9.89 |
| Uno |  | 0.002 | - | -1.37 | 1.91 | 1.78 | -7.92 | -12.55 | -8.42 |
| Sol |  | 0.003 | 0.18 | - | 2.42 | 2.96 | -7.22 | -9.76 | -3.34 |
| Onl |  | 0.08 | 0.07 | 0.02 | - | -0.12 | -8.22 | -13.77 | -8.11 |
| Par |  | 0.06 | 0.09 | 0.007 | 0.90 | - | -9.04 | -12.82 | -8.05 |
| Aso |  | <0.001 | <0.001 | <0.001 | <0.001 | <0.001 | - | -7.19 | 2.24 |
| Cop |  | <0.001 | <0.001 | <0.001 | <0.001 | <0.001 | <0.001 | - | 9.98 |
| Int |  | <0.001 | <0.001 | 0.003 | <0.001 | <0.001 | 0.04 | <0.001 | - |
